# The Association Between Depressive Symptomology, Psychological Burden Related to COVID-19, and Engagement in Physical Exercise Among College Students

**DOI:** 10.3389/fpsyt.2021.741964

**Published:** 2021-10-01

**Authors:** Zahir Vally, Mai Helmy

**Affiliations:** ^1^Department of Clinical Psychology, United Arab Emirates University, Abu Dhabi, United Arab Emirates; ^2^Wolfson College, University of Oxford, Oxford, United Kingdom; ^3^Psychology Department, Faculty of Arts, Menoufia University, Shebin El-Kom, Egypt

**Keywords:** depression, burden, coronavirus, COVID-19, exercise

## Abstract

**Introduction:** The coronavirus (COVID-19) pandemic has resulted in substantial changes to the normalcy of daily life. Research conducted in Western Europe point to elevated levels of depression, rumination and worry as well psychological burden. These in turn impact the capacity of individuals to adhere to lockdown measures and health-protective behaviours. Investigations of these pandemic-related mental health constructs in the Middle East appears sparse. Moreover, there is an immense need to investigate the potential for simple strategies that might be used by individuals whilst in lockdown to combat the onset of mental health difficulties. Regular physical exercise may prove valuable in this regard.

**Objective:** To investigate the potential mediational role of engagement in physical exercise on the association between depression and psychological burden related to COVID-19.

**Method:** A sample of 1,322 participants (m_age_ = 19.50 years, SD = 1.54) completed measures of depression and psychological burden related to COVID-19 and self-reported their frequency of physical exercise. Data were collected between February and May 2021 in Egypt and the United Arab Emirates.

**Results:** Analyses revealed statistically significant associations between depression and psychological burden as well as between elevated depression and reduced physical exercise. Mediation analyses in which the potential mediational role of physical exercise was examined were not significant.

**Conclusions:** Depression and psychological burden related to the pandemic appear to be prevalent in these Middle Eastern locations. Whilst physical exercise appears to be beneficial in combating depression, it does not appear to be a sufficient strategy for impeding the experience of psychological burden. Investigation of the contribution of additional strategies is required.

## Introduction

The coronavirus (COVID-19) pandemic which commenced in 2019 and has since remained with us and at times, intensified, has precipitated a number of necessary changes to the normalcy of society. At the time of writing this paper (July 2021), 188 million global cases of infection have been confirmed, whilst approximately 4 million people have died from coronavirus-related causes (1). In an attempt to retard the rapid spread of the disease, governments across the globe implemented strategies and instituted measures that significantly impacted the daily lives of their residents, most notably amongst these were the closure of public spaces, mandatory mask-wearing and social distancing (2). The latter, in particular, which was also accompanied by “stay-at-home” orders were particular taxing for many individuals and has come to be associated with documented increases in loneliness, mental health difficulties and, in some cases, suicide (3).

The psychological reactions of individuals to the experience of living with the pandemic appear to vary quite substantially. Some do indeed experience the pandemic, and the impact to daily living that it has precipitated, as an onerous task. For these individuals, frustration, worry about one's health and the future, anxiety, and fear of the uncertainty that pervades present life may be likely (3–5). For others, however, a more adaptive response appears possible. Some individuals have reported being able to maintain a semblance of a “normal life” that closely resembles their typical daily routine before the onset of the pandemic (6). These divergent methods of coping are noteworthy given that the manner in which individuals perceive and consequently elect to cope with the pandemic appears to measurably impact their psychological and physical health (3). Additionally, individuals' perception of the pandemic also appears to impact their compliance with the recommended health-protective behaviours and government mandates (e.g., mask-wearing) (7). This is an issue that has become an important public health concern during the pandemic as some segments of society appear to be more prone to non-compliance with government measures (2), and thus have placed their communities at risk of further infection. Compliance is only likely to be successful if it is widespread and consistent across societies.

Given these factors, it is imperative that researchers direct their attention towards the investigation of factors that might impact the degree of psychological burden caused by the pandemic. A thorough understanding of these factors could inform the development of strategies to identify individuals at risk of elevated levels of psychological burden, exploration of strategies targeted at its alleviation, the promotion of overall psychological health and potentially, as a secondary consequence, the promotion of greater compliance with health-protective and government-mandated preventive measures.

### Depression and COVID-Related Psychological Burden

Recent research has contended that the rate of depressive symptomology, particularly amongst the young adult population, can be expected to rapidly rise during the COVID-19 pandemic (8, 9). This contention is based on the documented evidence of a clear increase in the incidence of depressive disorders over the preceding decade (10) as well a burgeoning but preliminary body of evidence that has reported substantial rates of depressive symptomology amongst college students. This research has shown that the pandemic has precipitated incremental growth in the prevalence of depressive symptoms across all clinical levels, mild, moderate and severe presentations of the illness, and that these difficulties have been most intense when countries have been in states of complete lockdown (11, 12). The results of population-based studies further suggest that the adverse psychological consequences associated with the pandemic have indeed compounded over time. For example, in Switzerland, a study that included 10,472 participants from the general public found a prevalence rate of moderately severe and severe depressive symptomology of 9.1% during the first wave, and this proceeded to multiply to 11.7% during the lockdown that shortly followed, and this further rose to 18% during the second instituted lockdown (13, 14).

Depression is characterised by feelings of hopelessness, worthlessness, helplessness, anhedonia, and a sense that one's life is out of one's control (15). Individuals with depression also tend to ruminate about the current state of their lives and about what they typically perceive to be an uncertain future (16). These individuals tend to employ maladaptive strategies to cope with their lives, are likely to be socially withdrawn, experience excessive worry, and may present with suicidal ideation (17, 18). Given the nature and content of these depressive symptoms, it is plausible that individuals with a major depressive disorder diagnosis, or perhaps those from the general public with some depressive symptomology may be more likely to experience greater psychological burden as a result of the COVID-19 pandemic. Psychological burden related to COVID-19 has been defined by Brailovskaia et al. (19) as a sense of being overwhelmed by the pandemic, specifically, by the imposed restrictions to the experienced normality of one's life and the loneliness accompanied by social distancing measures. Moreover, a related construct to this definition is the inevitable lack of control of one's own life and of one's future that some individuals may experience whilst navigating the pandemic. Preliminary research has already begun to emerge that demonstrates a significant relationship between depression and perceived burden related to COVID-19. This is evident amongst individuals who have previously been hospitalised for a COVID-19 related illness and their family members (20), and amongst individuals employed as healthcare practitioners (21).

### Physical Exercise and COVID-Related Psychological Burden

A wealth of research demonstrates that engagement in regular physical exercise holds substantial benefits for individuals' physical and mental health. It reduces depressive symptoms, is beneficial for individuals with addictive tendencies, and appears to moderate the potentially deleterious impact of traumatic experiences (22–27). Physical exercise builds individuals' resilience to manage daily stress, promotes the development of positive emotional states, enhances self-efficacy, builds self-esteem, promotes a greater sense of self-control (28). Moreover, individuals who engage in regular physical exercise tend to be more flexible in response to daily stressors and are more likely to employ adaptive coping strategies when managing situations characterised by a degree of uncertainty (29, 30). Given these findings in relation to the positive effects of physical exercise, it is plausible that engagement in regular physical exercise may serve as a protective mechanism against the potentially deleterious effect of psychological burden precipitated by the coronavirus pandemic; in other words, individuals who report engagement in regular physical exercise will evidence lower psychological burden. Additionally, given the emerging evidence base which suggests that elevated levels of depression have become highly prevalent amongst individuals during the COVID-19 pandemic (8, 9, 11, 12, 19), physical exercise may also diminish the association between depression and psychological burden related to COVID-19.

There is some evidence to support the validity of this hypothesis, both in relation to the COVID-19 pandemic as well as during previous epidemics. Ahmed et al. (31) conducted a systematic review and meta-analysis of the long-term clinical outcomes of patients who had been hospitalised during the previous outbreaks of severe acute respiratory syndrome and Middle East respiratory syndrome. The authors report an overall prevalence rate for depression of 33% at 6 months post-discharge and this was associated with a reduced capacity for engagement in exercise. Brailovskaia et al. (19) report similar findings from a cross-national examination that included samples from Germany, Italy, Russia and Spain of the association between depression, exercise and psychological burden. It is reported that depression and psychological burden were significantly and positively associated and, moreover, engagement in physical exercise buffered this association allowing individuals to adapt to the demands of lockdown measures—this was evident across all the sampled countries (19). Additionally, Violant-Holz et al.'s (32) systematic review of the effect of physical exercise on psychological health reveals that exercise of sufficient intensity and duration diminishes psychological tension and improves mental stability.

Thus, the aim of the present study was to investigate the association between depressive symptoms, psychological burden related to COVID-19, and physical exercise. To investigate this aim, we proposed the following hypotheses: depressive symptoms should be positively associated with psychological burden (Hypothesis 1a); depressive symptoms should be negatively associated with physical exercise (Hypothesis 1b); psychological burden should be negatively associated with physical exercise (Hypothesis 1c); and physical activity should mediate the association between depressive symptoms and psychological burden related to COVID-19 (Hypothesis 2).

## Method

### Procedure and Participants

Ethical permission for the conduct of this study was obtained from the Institutional Review Boards of both the United Arab Emirates University and Menoufia University. We recruited participants in two different national contexts, Egypt and the United Arab Emirates (UAE). The study was advertised in the classes taught by the two researchers during the Spring semester of 2021. This represented a potential sampling frame of ~2,000 participants across the two research sites. In some classes in the UAE, students were offered course credit for participation in the study. This practise is customary in this location where students who are completing research-based courses participate in the studies of faculty members. Students were also asked to promote the study in their social circles. Advertisements were also placed on social media accounts typically used by the students at these two institutions. This process of recruitment resulted in a total of 1,322 participants (m_age_ = 19.50 years, SD = 1.54) who responded to advertisements to complete the online-administered surveys. Students who elected to participate accessed the electronic survey using a link that was included in the promotional material for the study. The link led to a consent form outlining the rights of participants and the responsibilities of the researchers. Clicking ahead to access the survey was indicative of the provision of consent.

With regard to the composition of the sample, participants were primarily students recruited from the student bodies of the researchers' universities (96.4%), a smaller minority of the sample were employed (2.6%), and the remaining few participants were unemployed (1%). The sample also tended to be predominantly female (75.4%) and single (90.6%). The total sample was comprised of 1,036 Egyptian participants and 286 participants from the UAE. Whilst most of the sample consisted of Egyptian participants (78.4 vs. 21.6% for the Egyptian and UAE participants, respectively), the proportional values of the distribution for all the demographic variables were not significantly different between the two country-stratified subsamples. These comparative values are illustrated in Table 1. Data were collected from February to May 2021.

**Table 1:** Descriptive statistics for all demographic variables including physical exercise (total sample and stratified by country).

	**Total *n* = 1,322**	**Egypt *n* = 1,036**	**UAE *n* = 286**
Age M (SD)	19.50 (1.54)	19.45 (1.43)	19.69 (1.88)
Gender (Female %)	75.4	74.1	80.1
**Marital status %**
Single	90.6	91.0	89.2
In relationship	7.0	6.6	8.4
Married	2.4	2.4	2.4
**Occupation %**
Student	96.4	96.7	95.1
Employed	2.6	2.2	4.2
Unemployed	1.0	1.1	0.7
**Physical exercise frequency %**
0 Never	34.6	35.1	32.5
1 Once a month or less	19.4	18.2	23.8
2 Two to four times a month	20.0	19.6	21.3
3 Two to three times a week	14.8	15.3	12.6
4 Four times a week or more	11.3	11.7	9.8

*M, mean; SD, standard deviation; UAE, United Arab Emirates*.

### Assessment Measures

#### Depression

We assessed depressive symptoms using the depression subscale of the Depression Anxiety Stress Scales 21 (DASS-21) (33). Participants respond to the seven items of the depression subscale using a 4-point Likert scale that ranges from 0 (did not apply to me at all) to 3 (applied to me very much or most of the time). Example items include “I felt that life was meaningless” and “I couldn't seem to experience any positive feeling at all”. Scores to the seven items are summed with higher cumulative scores indicative of greater severity of depressive symptomology. The DASS-21 is a widely used measure for the assessment of depressive and anxious affect. It has been shown to be highly reliable and valid. In particular, the validated Arabic version, employed in this study, has been demonstrated to be theoretically coherent and to possess adequate construct, convergent, and discriminant validity (34–36). It has been shown to be suitable for use across a wide variety of clinical contexts including with a college-aged population (37). Internal consistency for the Arabic version has ranged from 0.88 to 0.93 (34–36). Cronbach's alpha in the current study was similarly high (α = 0.81).

#### Psychological Burden

The degree of experienced psychological burden related to COVID-19 was assessed using the measure developed by Brailovskaia et al. (19) which consists of 6 items that are rated on a 4-point Likert scale from 0 (I do not agree) to 3 (I totally agree). Example items include “I feel socially isolated” and “I am burdened by the current social situation”. Higher cumulative scores across the 6 items are indicative of greater psychological burden. The measure has previously produced average to good internal consistency across a number of subsamples in a multi-country study (α = 0.65–0.77) (19). In the present study, Cronbach's alpha was 0.74, indicating satisfactory reliability.

#### Physical Exercise

We used a single item to measure the frequency of engagement in physical exercise [“How frequently did you engage in physical activity (e.g., jogging, cycling) in the last 12 months?”] to which participants responded using a 5-point Likert scale that ranged from 0 (never) to 4 (four times a week or more). This single item has previously been employed to measure the frequency of physical activity within the context of the COVID-19 pandemic and demonstrated to be a reliable and valid tool for doing so—specifically, evaluation of its psychometric properties has provided evidence that it performs as well as other brief physical activity tools and objective measurements of physical activity levels (19, 22, 27, 38).

### Data Analytic Strategy

**Figure 1:**
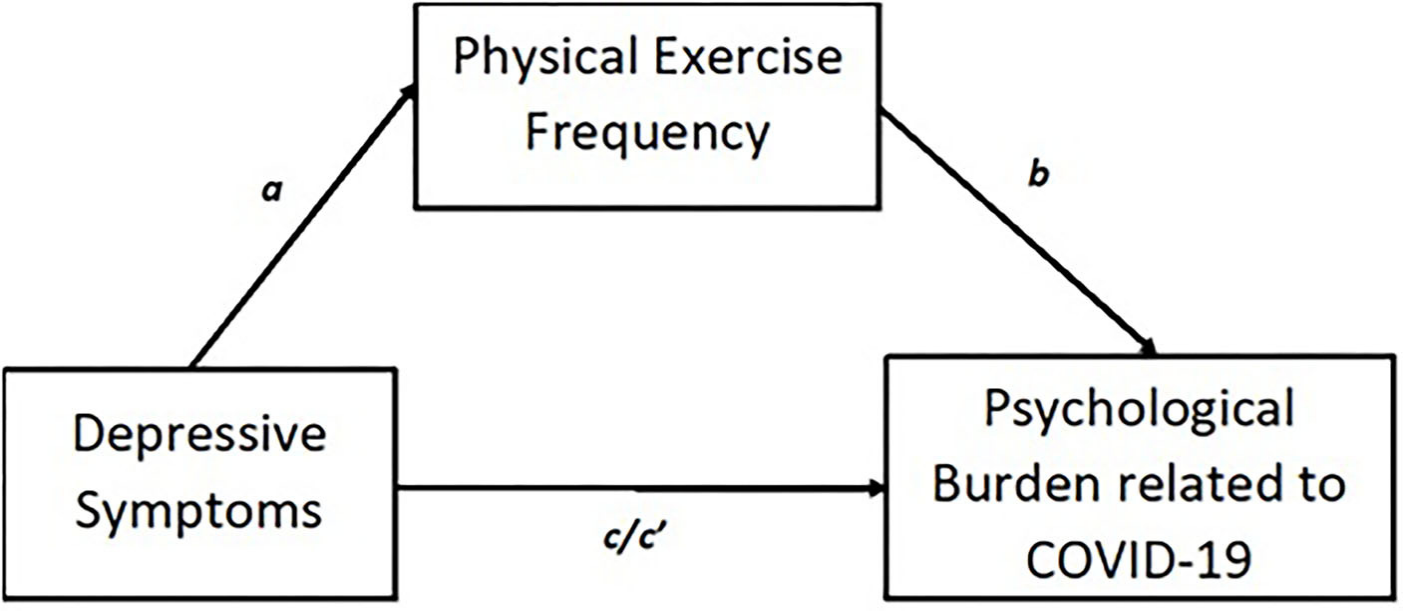
Proposed mediation model consisting of depressive symptoms as the predictor, physical exercise as the proposed mediator, and psychological burden as the outcome. a, path of predictor to mediator; b, path of mediator to outcome; c, path of predictor to outcome without mediator; c', path of predictor to outcome with inclusion of the mediator.

Descriptive analyses are reported using means and standard deviations or counts and associated percentages, as appropriate. First, we examined the association between psychological burden related to COVID-19, depressive symptoms, and physical activity by computing bivariate correlations, expressed as Pearson's correlation coefficient. Second, a pre-specified mediation model was examined that contained depressive symptoms as the predictor variable, physical activity as the potential mediator, and psychological burden as the outcome variable. Age and gender were included as covariates in the tested model. Figure 1 illustrates the proposed model. The relationship between depressive symptoms and physical activity is illustrated as path a while path b denotes the relationship between physical activity and psychological burden. The indirect effect (denoted as ab) is represented by the combined effect of paths a and b. The total effect illustrative of the association between depressive symptoms and psychological burden is denoted as path c, whilst the relationship between depression and psychological burden after inclusion of the proposed mediator, physical activity, is denoted as path c' (the direct effect). This mediational relationship was assessed using the bootstrapping procedure (10,000 samples) that results in bootstrapped confidence intervals (95%CI). We conducted mediation analyses following this procedure in both the overall sample as well as for each of the included countries. Analyses were conducted using SPSS 26 and the mediation analyses were conducted using the PROCESS macro version 3.5 (www.processmacro.org/index.html) (39). Statistical significance was set at *p* < 0.05 for all analyses.

## Results

Tables 1, 2 illustrate descriptive statistics for each of the study variables. Table 2 also presents the bivariate correlations between the three primary variables, depression, psychological burden, and physical activity. In both tables, results are included both for the overall sample as well as for each of the country-specific sub-samples. In the total sample, depression was significantly and positively associated with psychological burden (*r* = 0.58, *p* < 0.001) and negatively correlated with exercise frequency (*r* = −0.08, *p* = 0.003). A similar pattern of association between the variables was evident in the Egyptian sample. In the UAE sub-sample, psychological burden was similarly positively associated with depression and, while exercise frequency produced a negative pattern of association, this correlation was not statistically significant.

**Table 2:** Descriptive statistics and bivariate correlations between the three primary study variables (total sample and stratified by country).

	**M (SD)**	**Min–Max**	**2**	**3**
**Total sample n** **=** **1,322**
1 Depression	7.23 (3.88)	0–21	0.58^**^	−0.08^**^
2 Psychological Burden	7.85 (3.69)	0–18	-	−0.04
3 Exercise Frequency	1.49 (1.38)	0–4	-	-
**Egypt n** **=** **1,036**
1 Depression	7.18 (3.91)	0–21	0.59^**^	−0.09^**^
2 Psychological Burden	7.83 (3.71)	0–18	-	−0.05
3 Exercise Frequency	1.50 (1.40)	0–4	-	-
**UAE n** **=** **286**
1 Depression	7.38 (3.79)	0–21	0.57^**^	−0.06
2 Psychological Burden	7.94 (3.65)	0–18	-	−0.01
3 Exercise Frequency	1.43 (1.32)	0–4	-	-

*M, mean; SD, standard deviation; UAE, United Arab Emirates; Min, minimum; Max, maximum;*

** *p < 0.001 (2-tailed)*.

The mediation results were similar across the total sub-samples and both country-specific subsamples. Specifically, the total effect of depressive symptoms on psychological burden was significant and this relationship remained statistically significant after including physical exercise in the model, however, the coefficients (c and c') were almost identical suggesting that the included mediator contributed very little to the association between these two variables. This was confirmed by a nonsignificant indirect effect (the product of a and b) (see Table 3).

**Table 3:** Estimated coefficients of the tested mediation models for the total sample and stratified by country (outcome: psychological burden).

	**Total effect**			**Direct effect**			**Indirect effect**		
	**c**	**SE**	**95%CI**	**c'**	**SE**	**95%CI**	**ab**	**SE**	**95%CI**
**Total sample**
Burden	0.556	0.021	0.514–0.597	0.556	0.021	0.515–0.598	−0.001	0.002	−0.005–0.003
**Egypt**
Burden	0.557	0.024	0.511–0.604	0.558	0.024	0.511–0.605	−0.000	0.002	−0.005–0.005
**UAE**
Burden	0.549	0.047	0.457–0.642	0.551	0.047	0.459–0.653	−0.002	0.004	−0.012–0.006

*SE, standard error, 95%CI, confidence interval computed bootstrapping at 10,000 samples, c, relationship between depressive symptoms and psychological burden, c', relationship between depressive symptoms and psychological burden with the inclusion of physical activity in the model, ab, combined effect of paths a and b*.

## Discussion

This study examined the association between depressive symptomology and psychological burden related to COVID-19. Moreover, we sought to investigate the potential mediational role of engagement in physical exercise on this association. Previous research has demonstrated that, within the context of the COVID-19 pandemic, experienced psychological burden contributes substantially to deteriorating physical and mental health and reduces individuals' level of compliance with government mandates and health-protective behaviours (2–6). Therefore, the investigation of potential strategies that might mitigate the experience of psychological burden may have a measurable impact on the lives of individuals dealing with the mental health consequences of the restrictive measures imposed by the pandemic.

In confirmation of hypothesis 1a, our data revealed an association between depressive symptoms and psychological burden. The various measures implemented by governments to restrict the spread of the coronavirus, particularly in the Middle East, were experienced as being particularly restrictive to the lives of residents (40, 41). The lockdown measures and requirements for spatial distancing placed an enormous and onerous burden on the freedom with which individuals could live their typical lives. This, in turn, precipitated a range of mental health difficulties for many, the prevalence of which appears to have been especially pronounced amongst individuals with pre-existing mental health difficulties (11). Individuals with depression tend to experience low self-efficacy and are prone to ruminate and worry, factors that may contribute to elevated levels of experienced psychological burden when navigating an uncertain situation such as the pandemic. This demonstrated association between depression and psychological burden in our sample is therefore expected and in concurrence with samples from elsewhere in the world (8, 9, 11, 19).

A wealth of literature points to the protective effect of engagement in physical exercise for the effective management of mental health difficulties (21, 42, 43). It appears to diminish the experienced impact of negative life events and promotes the development of higher levels of resilience which individuals employ in the successful management of such events (15, 21). The coping strategies that individuals use in managing difficulties tend to be more adaptive and promote the development of a flexible approach towards uncertainty (29, 30). This is an important consideration and is especially relevant within the context of the present pandemic. The rules governing social distancing, working arrangements, travel, and the period of validity of negative COVID-19 test results for accessing the outside world are constantly in flux with amendments being made by governments in response to the changing nature of the pandemic. Therefore, we predicted that an increased frequency of engagement in physical exercise during the lockdown would be negatively associated with both depression and psychological burden. Analyses of our data confirmed a statistically significant and negative relationship between depressive symptoms and physical exercise (in confirmation of hypothesis 1b), but a similar relationship was not evident when examining psychological burden (hypothesis 1c was therefore not confirmed). Moreover, hypothesis 2 was also not confirmed. Despite significant total and direct effects, of the relationship between depression and psychological burden, engagement in physical exercise did not significantly mediate this relationship.

Therefore, our results suggest that individuals with depression do indeed appear to experience greater degrees of burden during the pandemic and may therefore be more likely to experience the range of deleterious effects to result from this, such as amplified depressive symptoms, elevated worry and rumination, and greater difficulty adhering to government-mandated lockdown measures. However, our results also indicate that a physical exercise regimen may not be sufficient to assist these individuals in managing their mental health difficulties. This is in contradiction to research conducted across Western Europe in which researchers suggested that, for the individuals whom they sampled, regular physical exercise enhanced self-efficacy and promoted as sense of self-control under uncertain circumstances which in turn resulted in the pandemic being experienced as less burdensome (19). This was not the case for the individuals in our sample.

The following potential explanations for the nonsignificant result are proposed. First, residents in the Gulf nations (e.g., the UAE, Qatar, Kuwait, and Bahrain) tend to live relatively sedentary lifestyles and consume diets that are high in fat and saturated sugars, this is both the result of the exorbitantly hot weather as well as the prevalence of an overt consumerist culture that has resulted from the region's economic success (44). This is evident in our sample in which the vast majority of participants (~75%) reported engaging in physical exercise less than a handful of times per month. A second potential explanation relates to what Marashi et al. (45) refer to as “a mental health paradox” in relation to the COVID-19 pandemic. They report in their large-scale analysis of Canadian respondents that the mental health burden associated with the pandemic served as a motivator to engage in physical exercise for some, and, in these cases, an appreciable benefit was evident, but for others, their experienced burden served to inhibit their engagement in exercise (45). This highlights an important consideration for the present study, specifically, that the association between exercise and mental health outcomes may not necessarily be straightforward. There may be a range of additional variables that potentially mediate and moderate this relationship (e.g., personality, resilience, fortitude), variables that were not assessed on this occasion.

### Limitations

This study's conduct is highly relevant and timely given that the pandemic appears likely to continue and the frequent reports of the substantial surge in mental health difficulties that have resulted from the pandemic continue to mount (13, 14, 20, 21). Despite this significant advantage, this study holds a number of limitations that should be borne in mind. First, despite the finding of a relationship between depression and psychological burden, the cross-sectional nature of our design points to an association but we cannot draw any conclusion about the nature of causality. Future longitudinal designs would enable greater clarity about this question and, in particular, about the causal relationship between increased frequency of physical exercise and reduced mental health difficulties.

Second, our findings are not generalizable across the complete spectrum of society. Our sample was relatively young and female in its composition. While the sample in our study is demographically different from previous investigations that have tended to be European and Caucasian in its composition and thus contributes an understanding about the phenomenon amongst an Arab population, our findings relate directly to a college-aged young adult population. These findings may only be applicable to a young adult and female population for whom it has been reported that depressive symptoms occur more prevalently (6, 10). Future studies should endeavour to include a more varied sample in terms of age and gender.

Third, given our implementation of this study across two different national contexts, the nature of the COVID-19 situation in these two countries were somewhat different at the points of data collection. In the UAE, strict lockdown measures were in place with curfews, limitations on movement, and work-from-home orders with associated fines instituted for violations of the mandates, whilst in Egypt, a more relaxed approach to the management of the pandemic was adopted. Despite this though, the scores on all variables and their computed effects in all analyses appeared to be relatively comparable with no significant differences between countries.

Finally, to measure the frequency of engagement in physical exercise, we employed a previously used measure however the self-report nature of this instrument is potentially problematic. Participants may have misrepresented their behaviour as a result social desirability or not of, recall bias. Moreover, given that we used a single item measure, it did not allow for a more nuanced measurement of participants' physical activity beyond their self-reported frequency. For example, the types of activities engaged in, the duration of sessions, and exercise intensity levels may variously impact the potential effect of physical activity on both depression and psychological burden, and, in turn, the mediational effect exercise may have on the relationship between them. This is a sensible hypothesis given preliminary research that has recently emerged from China in which the association between physical exercise and mental health differed according to the type of activity engaged in and the typical duration of the session (43). This is also especially relevant when considered in relation to the findings of Chekroud et al.'s (46) nationwide examination of the association of physical activity with mental health burden in the United States. This study reports that while physical activity was indeed associated with self-reported mental health status, increased frequency was not always necessarily beneficial. Thus, it is our recommendation that future studies attempt to employ objective measurements of physical exercise if this is feasible and appropriate or indeed to expand the dimensions of the construct to include measurement of type, duration, and intensity of physical exercise.

### Conclusion

In conclusion, our results provide evidence confirming the extent of the prevalence of depression and psychological burden related to the COVID-19 pandemic amongst samples from the Middle East—a target of research that has, thus far, rarely been examined in the literature. While our results also suggest that the increased frequency of physical exercise may not be sufficient to impede the negative effects of these mental health difficulties in this sample, it highlights the immense need to explore the applicability and effects of a wider and more varied array of interventions that might more successfully assist individuals struggling with the deleterious psycho-emotional effects of the pandemic.

## Data Availability Statement

The raw data supporting the conclusions of this article will be made available by the authors, without undue reservation.

## Ethics Statement

The studies involving human participants were reviewed and approved by Menoufia University's IRB and the Social Sciences Research Ethics Committee of the UAE University. The patients/participants provided their written informed consent to participate in this study.

## Author Contributions

ZV designed the study, collected data, conducted analyses, and wrote the draft of the manuscript. MH collected data, completed review of the manuscript, and edited accordingly. All authors contributed to the article and approved the submitted version.

## Conflict of Interest

The authors declare that the research was conducted in the absence of any commercial or financial relationships that could be construed as a potential conflict of interest.

## Publisher's Note

All claims expressed in this article are solely those of the authors and do not necessarily represent those of their affiliated organizations, or those of the publisher, the editors and the reviewers. Any product that may be evaluated in this article, or claim that may be made by its manufacturer, is not guaranteed or endorsed by the publisher.
